# The accuracy of dynamic wedge dose computation in the ADAC Pinnacle RTP system

**DOI:** 10.1120/jacmp.v5i4.1964

**Published:** 2004-11-24

**Authors:** H. Shao, X. Wu, C. Luo, S. Crooks, A. Bernstein, A. Markoe

**Affiliations:** ^1^ Radiation Oncology Department University of Miami, School of Medicine 1475 N.W. 12th Avenue Miami Florida U.S.A. 33136

**Keywords:** nonphysical wedges, radiation therapy

## Abstract

The nonphysical wedge is a modality that uses computer‐controlled jaw motion to generate wedge‐shaped dose distributions. There are Varian enhanced dynamic wedges (EDWs) and Siemens virtual wedges (VWs). We recently commissioned dynamic wedges on both Varian and Siemens LINACs. The beam data, acquired with a Wellhöfer chamber array and a Sun Nuclear profiler, are used for modeling in the ADAC Pinnacle system. As recommended by ADAC, only a limited number of beam data is measured and used for beam modeling. Therefore, the dose distributions of dynamic wedges generated by Pinnacle must be examined. Following the commissioning of the dynamic wedges, we used Pinnacle to generate a number of dose distributions with different energies, wedge angles, field sizes, and depths. The computed data from Pinnacle are then compared with the measured data.

The deviations of the output factor in all square and rectangular fields are mostly within 2.0% for both EDW and VW. For asymmetric fields, the deviations are within 3%. However, exceptions of differences more than 3% have been found in a larger field and large wedge combinations. The precision of the beam profiles generated by Pinnacle is also evaluated. As a result of this investigation, we present a scope of quality assurance tests that are necessary to ensure acceptable consistency between the delivered dose and the associated treatment plan when dynamic wedges are applied.

PACS numbers: 8753 Dq, 87.53.Xd

## I. INTRODUCTION

One of the advantages offered by modern LINACs is the ability to dynamically vary the jaw position during treatment to generate wedge‐shaped dose distributions. Using dynamic jaws to generate dose distributions equivalent to those produced by physical wedges placed in a static field was first proposed in the 1970s.^(^ ^1^ ^)^ Since then, the clinical implementation of the nonphysical wedge has been discussed by a number of investigators.^(^ ^2^ ^–^ ^9^ ^)^

Nonphysical wedges can generate discrete or continuous wedge angles up to 60°. Asymmetric wedges are available and are not centered on the isocenter line. The large range of field shapes and wedge angles introduces a new challenge for commissioning nonphysical wedges on modern treatment‐planning systems. The delivery of wedge‐shaped radiation therapy requires that the related components of the LINAC hardware and software be functioning correctly and that the RTP system has the ability to accurately model and calculate dose.

We recently commissioned and clinically implemented nonphysical wedges on four popular commercial clinic LINACs: the Varian 21EX and 2100C (Varian Oncology Systems, Palo Alto, CA) and the Siemens Primus KD and MD (Siemens Medical Systems, Concord, CA). The beam data are used for modeling in the ADAC Pinnacle system (ADAC Laboratories, Milpitas, CA). After commissioning, we compared the data from the Pinnacle RTP system with the measured data from the LINACs. The data demonstrated agreement within 2% for most symmetric and asymmetric fields, although there is a slight breakdown for some larger wedge angles with larger field sizes. After verification, we now confidently use nonphysical wedges our daily radiation treatment.

## II. METHODS AND MATERIALS

### A. Varian enhanced dynamic wedge

Nonphysical wedges are implemented on Varian LINACs as Varian enhanced dynamic wedges (EDWs). Varian uses the segmented treatment table (STT), which governs the position of the jaws with respect to the number of delivered monitor units (MUs). All treatment STTs are generated from a single energy‐dependent STT known as the golden STT for the Varian EDW method. The golden STT is a transmission table that produces a maximum wedge angle. The concept is that an intermediate sized wedge may be produced by the linear combination of the distribution from an open field and a maximum wedge field. For Varian machines the maximum angle is 60°, varying from 10° to 60° in seven discrete angles (10°, 15°, 20°, 25°, 30°, 45°, and 60°).

### B. The Siemens virtual wedge

Siemens introduced the virtual wedge (VW) to create wedge‐like dose distribution for their LINACs. The basic dosimetric principles of the Siemens VW are presented by van Santvoort.^(^ ^2^ ^)^ The jaw motion in the wedge direction can be analytically described by an exponential function ^(^ ^8^ ^)^:

(1) MU(Y)=MU(0)exp(cμY tanα),

where MU(*Y*)is the number of monitor units given at position *Y*, MU(0) is the number of monitor units at the center of the *Y*‐axis, *c* is the calibration factor, μ is the default mean linear attenuation coefficient, and α is the nominal wedge angle. One of the important features of the Siemens VW is that the wedge factor is designed to be 1.00±0.05. Unlike the Varian EDW, the Siemens VW gives the flexibility to plan with any wedge angle up to 60°. However, modeling on an arbitrary wedge angle is not supported by the ADAC Pinnacle RTP system.

For both Varian and Siemens LINACs, the dynamic wedge is only implemented in the direction of *Y*‐jaws with a maximum in *Y*‐jaw field size of 30 cm. For each *Y*‐jaw, the maximum open position is 20 cm, and the maximum to pass the central axis is 10 cm. Since only one jaw is employed in the wedge operation, the maximum range of the moving jaw is [−20,10]cm.

### C. ADAC Pinnacle RTP system

Pinnacle provides users with a flexible and optimal set of modeling and planning tools to incorporate dynamic wedges in the treatment‐planning process. The relative dose modeling is based on the open‐field model. The Varian or Siemens transmission array is generated using vendor‐specific parameters given the desired wedge angle, energy, and jaw setting of the beam. The Varian transmission array is the golden STT, while the Siemens transmission array is the exponential function of Eq. (1). The transmission array is then modified by vendor‐independent secondary effects, such as jaw transmission and head scatter. The dose computation is the combination of three processes: the open‐field model, the transmission array, and the secondary effects.^(^ ^10^ ^)^ Although the Siemens VW provides continuous wedge angle, ADAC Pinnacle only supports up to seven discrete wedge angles as defined by the Varian EDW.

### D. Measurements

Measurements were performed on two Varian 21EX, three Varian 2100C, one Siemens Primus KD, and one Primus MD. The dynamic nature of nonphysical wedges requires the use of a linear array of detecting devices. A Wellhöfer 3D scanning water phantom system (Wellhöfer Dosimetrie, Schwarzenbruck, Germany), equipped with an ion chamber array detector (Wellhöfer CA24), was used to scan the dose profiles. The chamber array detector has 23 ion chambers spaced 2 cm apart in a linear fashion and an additional reference chamber. The special resolution can be obtained up to 5 mm by shifting the chamber array by specific spacing. Data presented in this study were measured at 0.5 cm to 1.0 cm spacing, depending on the field sizes. We also used a diode array detector (Profiler Model 1170) manufactured by Sun Nuclear (Sun Nuclear Corporation, Melbourne, FL) for our measurements and for verification. The spatial resolution of the diode array is fixed at 0.5 cm. Both array detector systems have the ability to acquire the beam data for nonphysical wedges and offer similar accuracy. The diode array has the advantage of using a solid water phantom. Point‐by‐point measurements were also performed using an ion chamber to generate and to check the wedge dose profiles.

For the commissioning, the minimum nonphysical wedge profiles required by the ADAC Pinnacle RTP system are as follows: $5\times 5\;{\text{cm}}^{2}$, $10\times 10\;{\text{cm}}^{2}$, and $20\times 20\;{\text{cm}}^{2}$ at depths of 5 cm, 10 cm, and 20 cm. No depth dose profiles are required. The output factors were measured by a Farmer‐type ion chamber. Pinnacle requires the measurement of output on the depth of 10 cm only. All measurements were set at 100 cm source‐to‐surface distance (SSD). Usually we measure four or five wedge angles (10°, 15°, 30°, 45°, and 60°) to generate the seven discrete wedge angles. For data presented in this paper, the errors include the measurement error and the round‐off error from the calculation. We use MU=100 in the measurement and dose=100 cGy for dose calculation. The ADAC dose round‐off accuracy is about 0.5%.

## III. RESULTS

### A. Beam profiles

**Figure 1:**
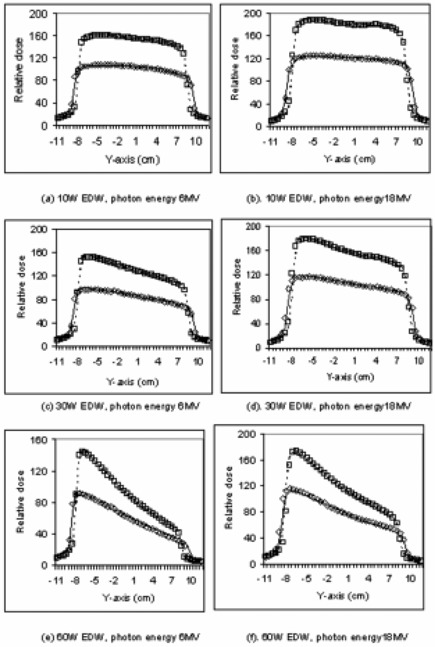
Comparisons of the EDW profiles generated by 21EX and Pinnacle; 100 cm SSD, 10×10cm field size. Displayed are the measured EDWs profiles at 20 cm depth (◊), at 10 cm depth (□). Also displayed as lines and dashed lines are EDWs profiles at 20 cm and 10 cm depths generated by ADAC Pinnacle calculations, respectively.

Beam profiles from nonphysical wedges were measured and compared with those from ADAC Pinnacle calculations. Figure 1 displays examples of dose profile comparisons for 6‐MeV and 18‐MeV photon beams of our Varian 21EX LINAC. The wedge angles are 10°, 30°, and 60°, and the measurement depths are 10 cm and 20 cm at field size of 15×15cm and 100 cm SSD. The measured data are from the Sun Nuclear diode array. In general, we see good agreement with 2% or 2 mm between measured and calculated profiles. However, in one of our Varian 2100 measurements, the maximum difference was as high as 8% in the toe side of the wedge. The output from ADAC data usually rises higher in the last 0.5 cm of the toe. As seen in Fig. 1, the areas under the wedges for measured and calculated profiles agree well, and the slight discrepancies seen in the edge of the toe are not considered to be a serious limitation of the system.

For the Siemens VW, we used point‐by‐point measurements to check the profiles. We measured the output at 5 cm from the wedge center. As listed in Table 1, all results agree within 2% between measured and calculated data. In Table 1, 1VW and 2VW indicate that the wedge orientation is formed by making *y*1 or *y*2 dynamic jaws. However, similar to the Varian EDW, Moued^(^ ^11^ ^)^ and Miften^(^ ^12^ ^)^ point out there may be a difference of up to 6% in the toe region on larger fields and larger wedge angles. This may be caused by overestimation by the VM model of the dose in the toe region for these field and wedge combinations.

**Table 1:** Calculated and measured dose on Siemens KD virtual wedge setup: field size = 15×15cm, depth=10cm

		6×		15×	
wedge	ISO	% diff at sup 5 cm	% diff at inf 5 cm	% diff at sup 5 cm	% diff at inf 5 cm
1VW15	100.0	0.6	1.0	0.2	0.1
2VW15	100.0	0.7	0.5	0.3	0.1
1VW30	100.0	1.1	0.7	1.3	0.7
2VW30	100.0	1.6	0.5	0.1	1.6
1VW45	100.0	0.3	0.8	0.5	0.0
2VW45	100.0	1.3	0.4	0.2	0.2
1VW60	100.0	0.1	1.6	1.8	0.1
2VW60	100.0	1.9	0.0	0.8	1.9

### B. Wedge factors of symmetric fields

Similar to the wedge factor for physical wedges, the wedge factor (WF) of the nonphysical wedge is defined as the ratio of the dose measured on the central axis of the wedge field $(D_{w}$) to the dose for the open field (*D*) with the same energy, depth, field size, and MUs:

(2) $${\text{WF}}_{\text{measured}}(\alpha ,d,s,E)=D_{w}(\alpha ,d,s,E)/D(d,s,E),$$

where α is the nominal wedge angle, *d* is the depth, *s* is the field size, and *E* is the nominal beam energy. The characteristics of an ideal dynamic wedge are a constant wedge factor as a function of field size, smoothly varying scatter factor with field size, the same percentage depth dose as open fields, and the ability to be used with asymmetric fields. As mentioned above, the Siemens VW has a wedge factor of close to unity. The WF of the Varian EDW, however, is a function of many parameters.

Table 2 shows the calculated and measured WFs for 6‐MeV and 18‐MeV 21EX photon beams. The WFs were measured by a Farmer ion chamber in a solid water phantom set at a depth of 5 cm with 100 cm SSD. As shown in the table, all errors between measured and calculated data are within 2%. The measured data show that the WFs are strongly dependent on, but change smoothly with, field size (*Y* side). These data are in agreement with results from other publications.^(^ ^5^ ^,^ ^6^ ^)^ For lower photon energies and a larger wedge angle, the WF changes more dramatically. The WF of the EDW changes little with the depth because the hardness effect introduced by the nonphysical wedge is minimal. We found that the WF changes less than 2% when depth changes from 5 cm to 20 cm.

**Table 2:** Wedge factors measured from 21EX and calculated from ADAC Pinnacle. All measurements are on d=5cm,100SSD

FS	5×5			10×10			15×15			20×20		
6×												
wedge	${\text{WF}}_{\text{ADAC}}$	${\text{WF}}_{21\text{EX}}$	%diff	${\text{WF}}_{\text{ADAC}}$	${\text{WF}}_{21\text{EX}}$	%diff	${\text{WF}}_{\text{ADAC}}$	${\text{WF}}_{21\text{EX}}$	%diff	${\text{WF}}_{\text{ADAC}}$	${\text{WF}}_{21\text{EX}}$	%diff
10	0.984	0.981	0.344	0.951	0.951	0.023	0.918	0.915	0.331	0.872	0.877	−0.570
15	0.977	0.971	0.592	0.929	0.927	0.170	0.875	0.880	−0.568	0.820	0.824	−0.540
20	0.962	0.961	0.087	0.907	0.902	0.552	0.836	0.843	−0.852	0.773	0.776	−0.380
25	0.955	0.947	0.797	0.880	0.880	−0.034	0.800	0.806	−0.744	0.727	0.729	−0.320
30	0.940	0.938	0.245	0.854	0.854	0.002	0.767	0.771	−0.503	0.681	0.684	−0.402
45	0.900	0.897	0.334	0.775	0.771	0.497	0.655	0.662	−1.062	0.553	0.556	−0.486
60	0.834	0.833	0.173	0.661	0.659	0.306	0.526	0.532	−1.161	0.424	0.422	0.503

18×												
wedge	${\text{WF}}_{\text{ADAC}}$	${\text{WF}}_{21\text{EX}}$	%diff	${\text{WF}}_{\text{ADAC}}$	${\text{WF}}_{21\text{EX}}$	%diff	${\text{WF}}_{21\text{EX}}$	${\text{WF}}_{21\text{EX}}$	%diff	${\text{WF}}_{\text{ADAC}}$	^WF^21EX	%diff
10	0.982	0.986	−0.359	0.963	0.964	−0.072	0.936	0.941	−0.549	0.909	0.913	−0.428
15	0.974	0.979	−0.520	0.946	0.946	−0.006	0.911	0.913	−0.229	0.870	0.874	−0.507
20	0.966	0.971	−0.565	0.929	0.930	−0.086	0.879	0.885	−0.604	0.833	0.837	−0.438
25	0.957	0.964	−0.699	0.905	0.911	−0.640	0.850	0.857	−0.824	0.794	0.801	−0.918
30	0.949	0.955	−0.612	0.890	0.891	−0.131	0.823	0.829	−0.765	0.763	0.763	0.047
45	0.918	0.924	−0.646	0.820	0.826	−0.689	0.729	0.737	−1.185	0.649	0.652	−0.406
60	0.875	0.874	0.114	0.724	0.732	−1.074	0.611	0.619	−1.361	0.518	0.521	−0.550
25	0.957	0.964	−0.699	0.905	0.911	−0.640	0.850	0.857	−0.824	0.794	0.801	−0.918
30	0.949	0.955	−0.612	0.890	0.891	−0.131	0.823	0.829	−0.765	0.763	0.763	0.047
45	0.918	0.924	−0.646	0.820	0.826	−0.689	0.729	0.737	−1.185	0.649	0.652	−0.406
60	0.875	0.874	0.114	0.724	0.732	−1.074	0.611	0.619	−1.361	0.518	0.521	−0.550

On the other hand, the Siemens VW, in principle, has a constant WF of unity. Results obtained from the Siemens machine are shown in Table 3. We found that the WF is generally 1.00±2%. With a large field size and a 60° wedge angle, the WF is as high as 1.07, usually coming from 6‐MeV beams. The divergence may be due to the additional scatter off the moving jaw as it travels across the beam portal. These effects are larger for large wedge angles and large field sizes compared with other setups.^(^ ^12^ ^)^

**Table 3:** Measured and calculated Siemens MD VW factors

6×,d=5cm	${\text{WF}}_{\text{primus}}$	${\text{WF}}_{\text{ADAC}}$	%diff	${\text{WF}}_{\text{primus}}$	${\text{WF}}_{\text{ADAC}}$	%diff
FS		5×5			15×15	
open	1.000	1.000	0.00	1.000	1.000	0.00
15w	1.000	0.992	0.81	1.004	1.000	0.40
45w	0.995	0.984	1.15	1.008	1.000	0.81

10×,d=5cm	${\text{WF}}_{\text{primus}}$	${\text{WF}}_{\text{ADAC}}$	%diff	${\text{WF}}_{\text{primus}}$	${\text{WF}}_{\text{ADAC}}$	%diff
FS		5×5			15×15	
15w	1.000	0.991	0.86	1.002	0.990	1.15
45w	0.989	0.983	0.65	0.987	0.990	−0.38

6×,d=15cm	${\text{WF}}_{\text{primus}}$	${\text{WF}}_{\text{ADAC}}$	%diff	${\text{WF}}_{\text{primus}}$	${\text{WF}}_{\text{ADAC}}$	%diff
FS		7×7			20×20	
20w	0.998	0.986	1.25	1.003	1.012	−0.87
50w	0.998	0.986	1.25	1.025	1.030	−0.52

10×,d=15cm	${\text{WF}}_{\text{primus}}$	${\text{WF}}_{\text{ADAC}}$	%diff	${\text{WF}}_{\text{primus}}$	${\text{WF}}_{\text{ADAC}}$	%diff
FS		7×7			20×20	
20w	0.998	0.989	0.95	1.006	1.007	−0.10
50w	0.982	0.978	0.41	0.990	1.007	−1.62

### C. Wedge factors of asymmetric fields

**Figure 2:**
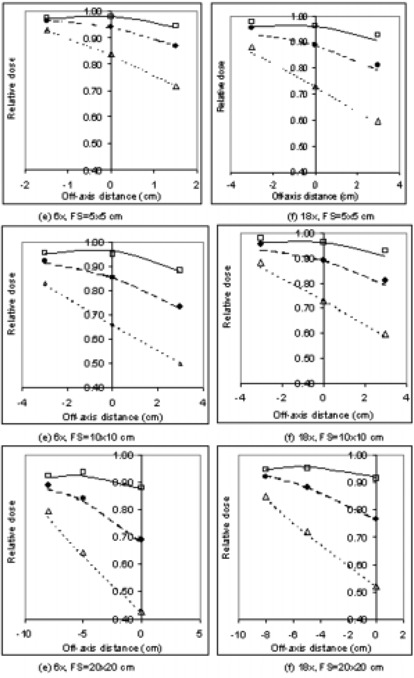
Asymmetric wedge factors versus off‐axis distance. Measured depth 10 cm, 100 cm SSD, on Varian 21EX. Displayed are the measured WF values for 10W (□), 30W (•), and 60W (Δ) dynamic wedges. Also displayed as lines are EDWFs generated by ADAC Pinnacle calculations.

Table 4 shows WFs from the Varian EDW results from asymmetric fields centered at different off‐axis positions. The field sizes demonstrated are 5×5cm, 10×10cm, and 20×20cm for 10°, 30°, and 60° wedges. All fields are asymmetric on *Y* jaws only. The displaced data in Fig. 2 demonstrate that the WF changes with off‐axis distance, which represents the distance from the central axis along the *Y* direction. The data demonstrate that the WF is not only field‐size dependent as in symmetric fields, but is strongly off‐axis dependent as well. Since the field is not symmetric, the WF is also dependent on the wedge orientation. In general, the agreement between measured and calculated data is within 2%. However, a difference of up to 3% to 4% has been found, which is larger than that found in the data for symmetric fields.

**Table 4:** Measured and calculated WFs for Varian 21EX asymmetric fields. All measurements at 100 SSD, d=10cm.

x=5	y1=1, y2=4			y=5			y1=4, y2=1		
	${\text{WF}}_{21\text{EX}}$	${\text{WF}}_{\text{adac}}$	%diff	${\text{WF}}_{21\text{EX}}$	${\text{WF}}_{\text{adac}}$	%diff	${\text{WF}}_{21\text{EX}}$	${\text{WF}}_{\text{adac}}$	%diff
W10	0.974	0.971	0.269	0.980	0.983	−0.260	0.942	0.939	0.349
W30	0.963	0.966	−0.314	0.940	0.939	0.118	0.869	0.871	−0.199
W60	0.929	0.929	0.057	0.837	0.833	0.539	0.715	0.716	−0.216

x=10	y1=2, y2=8			y=10			y1=8, y2=2		
	${\text{WF}}_{21\text{EX}}$	${\text{WF}}_{\text{adac}}$	%diff	${\text{WF}}_{\text{adac}}$	${\text{WF}}_{21\text{EX}}$	%diff	${\text{WF}}_{21\text{EX}}$	${\text{WF}}_{\text{adac}}$	%diff
W10	0.883	0.878	0.618	0.950	0.962	−1.225	0.957	0.950	0.735
W30	0.735	0.729	0.758	0.855	0.853	0.222	0.923	0.915	0.894
W60	0.500	0.502	−0.331	0.664	0.659	0.699	0.832	0.825	0.791

x=20	y1=2, y2=18			y=20			y1=5, y2=15		
	${\text{WF}}_{21\text{EX}}$	${\text{WF}}_{\text{adac}}$	%diff	${\text{WF}}_{21\text{EX}}$	${\text{WF}}_{\text{adac}}$	%diff	${\text{WF}}_{21\text{EX}}$	${\text{WF}}_{\text{adac}}$	%diff
W10	0.923	0.913	1.029	0.879	0.873	0.732	0.936	0.919	1.838
W30	0.888	0.873	1.750	0.688	0.682	0.940	0.836	0.825	1.340
W60	0.794	0.770	3.154	0.429	0.427	0.518	0.644	0.628	2.466

18×									
x=5	y1=1, y2=4			y=5			y1=4, y2=1		
	${\text{WF}}_{21\text{EX}}$	${\text{WF}}_{\text{adac}}$	%diff	${\text{WF}}_{21\text{EX}}$	${\text{WF}}_{\text{adac}}$	%diff	${\text{WF}}_{21\text{EX}}$	${\text{WF}}_{\text{adac}}$	%diff
W10	0.960	0.951	0.880	0.987	0.986	0.141	0.931	0.926	0.623
W30	0.949	0.945	0.476	0.955	0.951	0.380	0.879	0.873	0.765
W60	0.923	0.919	0.331	0.874	0.867	0.797	0.754	0.753	0.179

x=10	y1=2, y2=8			y=10			y1=8, y2=2		
	${\text{WF}}_{21\text{EX}}$	${\text{WF}}_{\text{adac}}$	%diff	${\text{WF}}_{21\text{EX}}$	${\text{WF}}_{\text{adac}}$	${\text{WF}}_{21\text{EX}}$	${\text{WF}}_{\text{adac}}$	%diff	
W10	0.929	0.907	2.373	0.964	0.962	0.195	0.984	0.962	2.226
W30	0.811	0.794	2.135	0.890	0.888	0.213	0.955	0.934	2.239
W60	0.597	0.585	1.957	0.731	0.730	0.153	0.882	0.864	2.093

x=20	y1=2, y2=18			y=20			y1=5, y2=15		
	${\text{WF}}_{21\text{EX}}$	${\text{WF}}_{\text{adac}}$	%diff	${\text{WF}}_{21\text{EX}}$	${\text{WF}}_{\text{adac}}$	%diff	${\text{WF}}_{21\text{EX}}$	${\text{WF}}_{\text{adac}}$	%diff
W10	0.945	0.945	0.061	0.914	0.916	−0.222	0.955	0.952	0.227
W30	0.919	0.923	−0.390	0.765	0.764	0.087	0.882	0.882	−0.061
W60	0.848	0.839	1.060	0.523	0.522	0.242	0.719	0.714	0.727

## IV. CONCLUSION

The implementation of nonphysical wedges into an RTP system provides clinics with an effective tool for treatment planning and radiation therapy treatment. The use of such computer‐controlled dose modulators requires accurate dose and wedge factor calculations.

After commissioning the Varian EDW and Siemens VW, the measured data were implemented in the ADAC Pinnacle RTP system. We tested thoroughly to compare the measured data calculation with the output data from the Pinnacle system. The dose verification results demonstrate that the ADAC Pinnacle RTP system can generate clinically accurate dose calculations for both the Varian EDW and Siemens VW.

## ACKNOWLEDGMENT

The authors would like to thank Dr. Hai Luo, Horton Medical Center, Middletown, NY, and Dong Meng, Wentworth Douglass Hospital, Dover, NH, for their support. The authors also thank Ms. Joyce Mosier for her editorial review.
